# Qualitative and Nutritional Improvement of Cereal-Based Foods and Beverages

**DOI:** 10.3390/foods10020338

**Published:** 2021-02-05

**Authors:** Antonella Pasqualone, Carmine Summo

**Affiliations:** Department of Soil, Plant and Food Science (DISSPA), University of Bari Aldo Moro, Via Amendola, 165/a, I-70126 Bari, Italy; carmine.summo@uniba.it

**Keywords:** functional foods, upcycling, byproducts, bioactive compounds, dietary fiber, new quality, pulses, insects, bread, pasta

## Abstract

The main directions of research aimed at nutritional improvement have to face either excesses or deficiencies in the diet. To this end, different strategies may be adopted, such as the reformulation of products, the introduction of functional ingredients, and the application of biotechnology to increase the bioavailability of bioactive compounds. These interventions, however, can alter the physico-chemical and sensory properties of the final products, making it necessary to achieve a balance between nutritional and quality modification. This Special Issue offers readers information on innovative ways to improve the cereal-based foods and beverages, useful for researchers and for industry operators.

Increased consumer awareness of the effects of food in preventing nutrient-related diseases and maintaining physical and mental well-being, has made nutritional improvement an important goal of the food and beverage industry, including the cereal sector. To this end, different strategies may be adopted, such as the reformulation of products, the introduction of functional ingredients, and the application of biotechnologies to increase the bioavailability of bioactive compounds. These interventions, however, can alter the physico-chemical and sensory properties of the final products, making it necessary to achieve a balance between nutritional and quality modification.

The Special Issue “Qualitative and Nutritional Improvement of Cereal-Based Foods and Beverages” collects 17 original research articles and one review aimed at exploring innovative ways to improve cereal-based foods and beverages, an old—if not ancient—group of products which are still on our table every day.

In these articles, a wide range of very different food products is considered, such as bakery products (including white bread, brown bread, durum wheat bread, tortilla, pizza base, muffins and biscuits), fresh and dry pasta, extruded sticks and instant flours, fortified blended foods and Sunsik, the latter being a traditional Korean beverage [1]. Cereal-based beverages, indeed, hold a long tradition and have become known for their sensory and health-promoting attributes [2].

The main directions of research aimed at nutritional improvement have to face either excess or deficiency in the diet. In the latter case, nutrient-rich foods with long shelf-life are needed to prevent malnutrition, whereas in developed countries it is mostly required to decrease the energy value, sucrose, salt, and increase dietary fiber content of foods to prevent obesity and nutrient-related chronic diseases such as cardiovascular disease, hypertension, and diabetes mellitus. The 2030 Agenda for Sustainable Development, adopted in 2015 by the United Nations, set the goals “*to end hunger, achieve food security and improved nutrition, and promote sustainable agriculture*” (Goal 2) and “*to ensure healthy lives and promote well-being for all at all ages*” (Goal 3), recognizing non-communicable diseases (NCDs) as a major challenge for sustainable development [3]. In addition, patients with obesity and other chronic underlying conditions are at particularly high risk of developing severe COVID-19 complications [4].

The most natural way to improve the nutritional profile of cereal-based foods is to use wholemeal flour, thus retaining all fiber and micronutrients of wheat caryopsis. Wholewheat flour is a valuable raw material, irrespective of the milling system (stone milling or roller milling) [5]. Higher contents of bioactives can be reached if purple wheat, whose debranning fractions are particularly rich in anthocyanins, is used [6]. Fermentation further improves the nutritional features of wholemeal flours. Fresh pasta prepared by mixing semolina with wholewheat sourdough shows higher content of free essential amino acids and phenolic compounds, lower phytic acid content, and higher antioxidant activity, than control pasta where non-fermented wholewheat semolina is used [7].

Another strategy consists in adding locally available ingredients to reformulate existing cereal-based foods, in order to increase the nutritional value while diminishing the risk of genetic erosion of the local crops and reduce imports. In this context, the leaf powder of *Moringa oleifera*, a plant originating in India and Africa, which is rich in proteins, minerals, and phytochemicals, has been proposed as an additional ingredient to improve the nutritional profile of white and brown bread [8] or fresh pasta [9]. The fortification with moringa leaf increases protein and iron content, but makes bread darker lowering the consumer acceptability, although with a minor impact on brown bread. The addition of moringa leaf powder to fresh pasta in the range 5–15% significantly increases the content of polyphenols even at the lowest percentage. Similarly, legumes can be used which are rich in proteins and complement the amino acid profile of cereal-based foods. Flour of Apulian black chickpeas, an autochthonous black-coated chickpea cultivated in Southern Italy, rich in anthocyanins, has been added to various bakery products [10], namely bread, “focaccia”—an Italian traditional bakery product similar to pizza [11], and pizza crust by substituting flour in the 10–40% range. The rheological properties of dough worsen, resulting in harder and darker final products. However, the nutritional features improve in terms of higher contents of fiber, proteins, and bioactive compounds, as well as higher antioxidant activity [10]. In pasta, the addition of chickpea and hemp flour, previously fermented and enzymatically treated, improves the nutritional profile and protein digestibility, and reduces the sensory drawbacks and the antinutrients (tannins, phytates and raffinose) [12].

Red quinoa or Taiwan djulis (*Chenopodium formosanum* Koidz.) can be used to develop sourdough bread (20% djulis sourdough and 80% wheat flour) [13], whereas germinated wheat, in combination with an extract of *Achyranthes aspera* and *Acanthopanax*, two plants used in Asian traditional medicine, has been proposed to fortify Sunsik, a traditional ready-to-drink Korean cereal-based beverage made of roasted brown rice, barley, adlay, oat, and black beans [1]. Amaranth (*Amaranthus hypochondriacus* L.) and flaxseed (*Linum usitatissimum* L.), instead, have been added to corn-based instant-extruded products, to meet the needs of nutritionally balanced ready-to-eat foods. The addition effectively increases lysine, polyunsaturated fatty acids, minerals, and fiber of the end-products [14]. Seed flour of *Brosimum alicastrum* Sw., a Mexican tree locally named “ramón,” characterized by high protein, dietary fiber, and micronutrient content, has been added to wheat tortillas improving the healthy features while keeping pliability unaltered, but with a browner color [15].

Insects are another valuable source of proteins which could be used to overcome the challenge of a more sustainable food chain while improving the nutritional profile of end-products. Meal of winged termites (*Macrotermes bellicosus*) has been added to biscuits prepared with sorghum and wheat flour [16]. Sorghum, which can be grown in tropical areas, makes this biscuit formulation viable in sub-Saharan areas, where protein-energy malnutrition is a major health concern. A significant increase of proteins, minerals, and amino acids is achieved, but biscuits become darker and less hard [16]. In the same geographic area, fortified blended foods (corn-soy blends or wheat-soy blends) are used to prepare viscous porridges in supplementary feeding programs [17]. These food aids often result in products too viscous for being fed to infants and young children, therefore it is needed to dilute them, lowering the nutritional value and energy density. The addition of cowpea has been proposed, also in combination with extrusion cooking, to obtain a porridge able to deliver the correct amount of nutrients at lower viscosities [17]. Extruded cooked products are rehydrated easily without cooking [18], which is important to save energy sources.

The use of waste and byproducts from the food industry, where upcycling is becoming increasingly important to improve sustainability, represents another mean for enhancing the nutritional and healthy features of cereal-based foods [19]. Coffee silverskin, a byproduct from the coffee industry rich in dietary fiber, proteins and bioactive compounds, has been used to produce extruded sticks based on cornmeal and amaranth flour [20], whereas almond skins, a byproduct of almond-based confectionery industry, rich in fiber and phenolic compounds, have been effectively considered as a functional ingredient in biscuits, which become more friable but darker [19].

Finally, to meet the needs related to western lifestyle, the production of muffins containing agave syrup instead of sucrose [21], and the reduction of the sodium content of bread by using a natural low-sodium sea salt [22], have been proposed.

To sum up, this Special Issue gives an interesting contribution to the field and offers readers information on several ways to innovate and improve the cereal-based foods and beverages, which can be useful both for researchers and for industry operators. In most cases, the reformulation with additional ingredients alters the sensory properties, therefore raising the need of communicating a “new quality” to consumers to explain that the differences with conventional counterparts are largely compensated by improved nutritional features.
